# *In situ* evaluation of contact stiffness in a slip interface with different roughness conditions using ultrasound reflectometry

**DOI:** 10.1098/rspa.2021.0442

**Published:** 2021-11

**Authors:** S. Fukagai, M. Watson, H. P. Brunskill, A. K. Hunter, M. B. Marshall, R. Lewis

**Affiliations:** ^1^ Railway Technical Research Institute, Tokyo, Japan; ^2^ Department of Mechanical Engineering, University of Sheffield, Sheffield, UK

**Keywords:** wheel–rail, ultrasound reflectometry, *in situ* measurement, contact stiffness, high-pressure torsion test, contact simulation

## Abstract

Understanding the dynamic condition of the interface between a railway wheel and rail is important to reduce the risks and consider the effectiveness of countermeasures for tribological problems. Traditionally the difficulty in obtaining accurate non-destructive interfacial measurements has hindered systematic experimental investigations. Recently, an ultrasound reflectometry technique has been developed as a direct observation method of a rolling–sliding interface; however, the topography dependence under the high contact pressures in a wheel–rail contact has not been clarified. For this reason, a novel *in situ* measurement of the contact stiffness using ultrasound reflectometry was carried out for three different levels of roughness. A contact pressure equivalent to that in a wheel–rail interface was achieved by using a high-pressure torsion test approach. The dynamic change of contact stiffness with slip was measured using ultrasound and the influence of roughness was investigated. The measured changes were validated using a newly developed numerical simulation, and mechanisms to explain the observed behaviour were proposed in terms of fracture and plastic deformation of the asperity bonds. These findings could help in understanding the traction characteristics for different roughness conditions and also assist in understanding damage mechanisms better, such as wear and rolling contact fatigue.

## 1. Introduction

The wheel and the rail play vital roles in rail operation, such as bearing the vehicle load, guiding the vehicle and transmitting the driving and braking forces. To achieve these roles, the wheel and the rail roll/slide against each other under extremely high contact pressure conditions. Due to the severe contact condition, the interface is the origin of a number of tribological problems during operation. For example, when contaminants, such as water, oil or fallen leaves, get into the interface, the interaction can lead to wheel spin and brake lock-up. Such significant slipping can cause not only performance problems in terms of delays and safety issues from over-running (past signals at danger or a station), but also thermal damage and abnormal deformation of wheel and rail [1,2]. Also, it is known that high friction coefficient and slip at curves could lead to severe wear and deformation of wheel and rail [3,4], high energy consumption [5] and wheel–rail noise [6,7]. Additionally, it increases the risk of a wheel climb derailment occurring [8–10].

To consider effective countermeasures for the problems mentioned above and to reduce the risks, it is important to understand the dynamic conditions in the interface. However, the difficulty in obtaining accurate non-destructive interfacial measurements has hindered systematic experimental investigations.

The use of pressure-sensitive films is one potential method to evaluate the contact area and pressure [11,12]. However, these films will change the tangential load due to their different frictional properties, and they act as a ‘gasket’ so change the load distribution in the interface. Practical implementation is also difficult because the film disintegrates under high pressure and shear between the wheel and the rail. A method using a fibre Bragg grating sensor which is embedded in the rail to evaluate the distribution of wheel–rail contact pressure has also been reported [13]. Although the contact pressure can be evaluated by measuring the strain in the rail in this method, there is difficulty in investigating the tribological phenomenon at the interface.

Recently, ultrasonic techniques have been used to observe the contact between wheel and rail [14–21]. Though there are spatial resolution limits and considerations of transducer positioning to ensure the sound waves reflect off the area of interest, this technique can be used to non-invasively and directly observe the contact. When an ultrasonic wave strikes the interface between the wheel and rail, it is partially transmitted and partially reflected. The proportion of the wave reflected depends on the stiffness of the contact [22,23]. This approach has been used to determine the contact pressure distribution in wheel–rail contacts and the influence of wear profile, roughness and surface defects on the contact patch [15,17]. Also, this actual distribution of the contact pressure could be applied to the simulation of wear and damage propagation with consideration of surface topography [24,25].

The authors have already investigated the influence of the topography on the friction behaviour between the wheel and rail in dry condition focusing on the mechanisms of flange climb derailments [26]. As a result, it was found that the initial topography affected the friction behaviour during running in. Though these results indicate that the dynamic evolution of the surface asperities relates to the friction behaviour, these findings were based on the surface investigation when the test was stopped intermittently. Some of the authors also investigated the relationship between the change in contact condition and the change in friction coefficient by scanning the contact area with a linear array ultrasonic transducer attached to a full-scale wheel–rail contact rig [27]. The friction coefficient tends to increase as the normal contact stiffness, evaluated from the echo amplitude of the ultrasound, increases. In order to understand this phenomenon in more detail, it is necessary to capture in real time how the topography of the interface changes from time to time. Recently, a fundamental study to investigate the interfacial condition with micro-periodic vibration using ultrasonic waves was reported [28]. This study showed that the reflection coefficient dynamically changed with the progress of the friction mode, such as static to macro-slip. However, the experiments were carried out under a 10 MPa contact pressure, which is much lower than the wheel–rail contact pressure, and the influence of topography has still not been investigated. If the ultrasonic technique could be applied to a slip interface which simulates the slip component of the wheel–rail interface, it will enable clarification of how the interfacial topography between the wheel and rail with extremely high contact pressure changes with frictional motion.

The aim of this work was to understand the influence of the roughness on the dynamic friction behaviour between the wheel and rail. To achieve a contact pressure equivalent to that in a wheel–rail interface, a high-pressure torsion (HPT) test approach was used. The HPT testing equipment is capable of applying horizontal relative motion (slip) to two surfaces in contact with each other, while achieving a high contact pressure. The contact area is large enough to evaluate the contact stiffness using ultrasonic waves. Tiny piezoelectric elements which generate the ultrasonic waves were bonded to one of the test specimens. Ultrasonic reflection from the interface was used to conduct *in situ* evaluation of the contact condition, particularly contact stiffness. Transient loading conditions and displacement were also measured during the test. Following these measurements, and the changes of contact stiffness with contact pressure, slip distances were reproduced numerically.

## 2. Methodology

### (a) High-pressure torsion testing equipment

**Figure 1.**
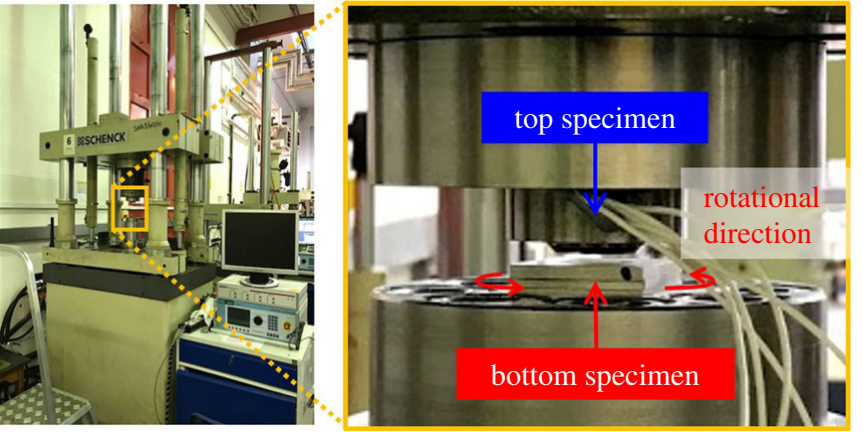
The appearance of the HPT equipment. (Online version in colour.)

Figure 1 shows the appearance of the HPT testing equipment. This equipment is capable of making contact between two specimens with a constant normal stress and then rotating the bottom specimen in the direction parallel to the contact interface [29,30]. It uses load cells for tension, compression and torque to measure the compressing load and the torque and uses a rotary variable differential transformer to measure the rotation speed. It has a maximum axial load (tensile and compression) of ±400 kN, an axial movable range of ±25 mm, a maximum torque of ±1000 Nm and a rotational movable range of ±40°.

**Figure 2.**
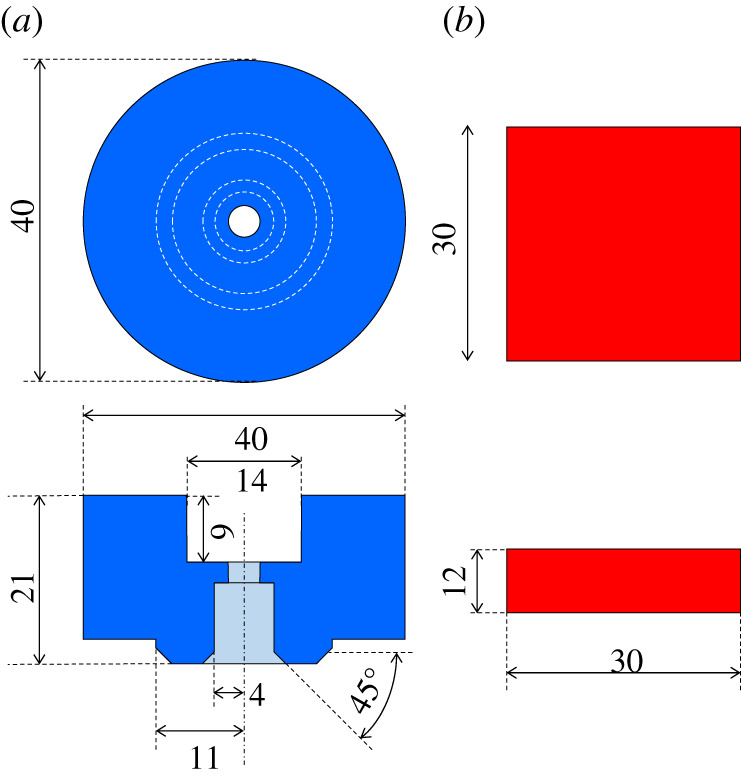
The initial design of specimens for the HPT test. (*a*) Top specimen and (*b*) bottom specimen. (Online version in colour.)

**Figure 3.**
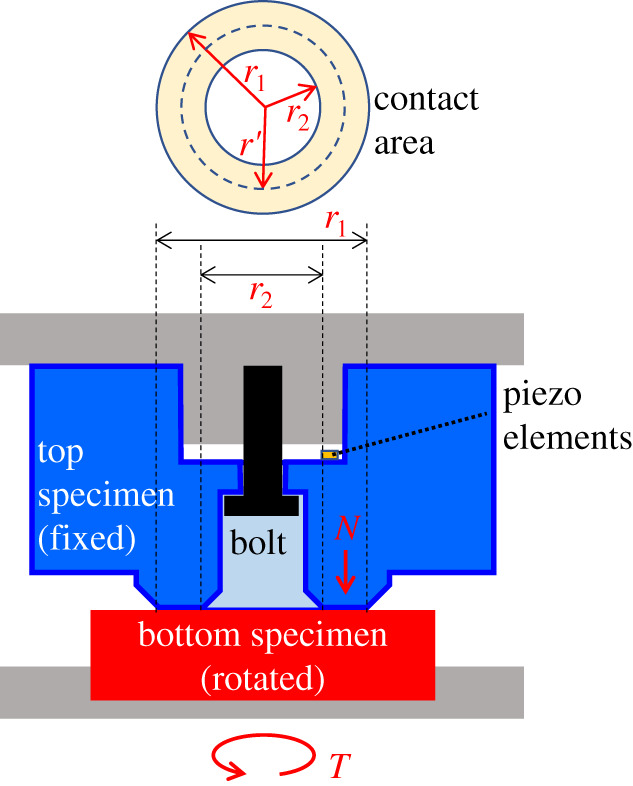
Schematic of the HPT testing equipment. (Online version in colour.)

Figure 2 shows the initial design of specimens for the HPT testing equipment [29]. Specimens are installed in the top and bottom parts of the equipment and a pair of the specimens is used as a unit. The top specimen is shaped in a cylindrical form. The bottom specimen is shaped in a rectangular solid form. Both specimens are reset by grinding, blasting or cutting the contact surface for each test. Therefore, although the height and other dimensions of the samples vary slightly from test to test, the contact area was measured for each test to define about the same contact pressure. Figure 3 shows a schematic of the HPT testing equipment. The contact shape can be seen as an annular shape. The annular contacts were used so that typical contact pressures could be achieved at the loading capacity of the apparatus. The test specimens are to be as parallel as possible so that a uniform pressure is obtained at each radius of the annular contact.

The relationship between the normal load *N* and the normal stress *σ_N_* is represented as in equation (2.1):

2.1 $$\sigma_{N}=\frac{N}{\pi ({r_{1}}^{2}-{r_{2}}^{2})}.$$

In equation (2.1), *r*_1_ and *r*_2_, respectively, represent the outer and inner diameters of the hollow circle of the contact area. Equation (2.2) shows the relationship between the torque *Tq* and the tangential load *T* [29]:

2.2 $$T=\frac{Tq}{2/3(({r_{1}}^{3}-{r_{2}}^{3})/{r_{1}}^{2}-{r_{2}}^{2})}.$$

In addition, the relationship between the tangential load *T* and the tangential stress *σ_T_* can be represented as in equation (2.3):

2.3 $$\sigma_{T}=\frac{T}{\pi ({r_{1}}^{2}-{r_{2}}^{2})}.$$

### (b) Ultrasound reflectometry

In this study, the contact stiffness was measured in three directions: normal and two tangential directions, slip direction and perpendicular to the slip direction, based on the intensity of the ultrasonic wave reflected from the contact interface. In the following, the principle of the evaluation of contact phenomena by ultrasonic reflectometry will be explained.

At the interface between materials with different acoustic impedances, only a part of a sound wave transmits at the interface and the rest of it is reflected back. The reflectivity for an ultrasound at an interface where the materials adhere to each other without any cavities, *R*, can be represented as in equation (2.4), and it varies depending on the difference in the acoustic impedances of the two materials:

2.4 $$R=\frac{z_{2}-z_{1}}{z_{2}+z_{1}}.$$

In this equation, *z*_1_ and *z*_2_ are the acoustic impedances of the materials in contact. The acoustic impedance is determined by the product of the density of the material and the acoustic velocity in the material. Therefore, when the acoustic impedances of the two materials in contact are the same and if the interface is hypothetically perfectly conformal, all the sound waves will transmit at the interface without any loss and no reflection occurs (*R* = 0). On the other hand, when materials with significantly different acoustic impedances, such as a gas and a solid, are in contact, sound waves are almost completely reflected (*R *≈ 1).

**Figure 4.**
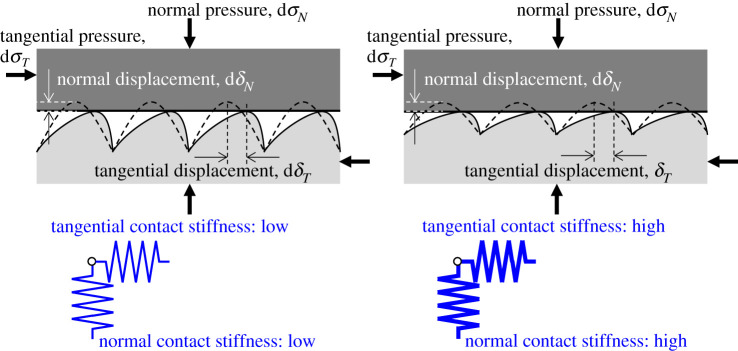
Schematic model of an imperfect interface with ideal asperities. Normal and tangential loads are simulated. (Online version in colour.)

Figure 4 shows a schematic model of an ideal asperity contact loaded in both the normal and tangential directions. The surface of an actual material is not perfectly flat, but has micro-asperities and undulations, so that when two bodies come into contact, an interface with air cavities is created. When the wavelength of the ultrasound is sufficiently larger than the cavity size at the interface, the proportion of the reflected wave also depends on the contact stiffness. The contact stiffness is a function of the number, size and approach of the contact points determined while considering the minute asperities [22]. Because the topographies of the surface change due to elastic and plastic deformation, the measured reflectivity changes as shown in figure 4 as load is applied. Therefore, it is possible to evaluate the contact condition at the interface by using the reflectivity of ultrasound.

Schoenberg [31] adopted the spring interface model to express the reflectivity of ultrasound in the following equation:

2.5 $$R=\frac{z_{2}-z_{1}-i\omega (z_{1}z_{2}/K)}{z_{2}+z_{1}-i\omega (z_{1}z_{2}/K)},$$

where *ω* and *K*, respectively, represent the angular frequency of the ultrasound and the contact stiffness. For the example in this article, the equation can be simplified as follows because it is assumed that the wheel and the rail are made of the same material (*z*_1_ = *z*_2_ = *z*):

2.6 $$|R|=\frac{1}{\sqrt{1+{\left(2K/\omega z\right)}^{2}}}.$$

Therefore, it is possible to evaluate the contact stiffness *K* if the reflection coefficient *R* can be determined in an experiment. Generally, the contact stiffness is defined as the normal or tangential stress generated when the relative distance between two surfaces in contact via surface asperities changes by a unit length in each direction as shown in figure 4. The contact stiffnesses for each direction are expressed as shown in the following equations:

2.7 $$K_{N}=\frac{\text{d}\sigma_{N}}{\text{d}\delta_{N}}$$

and

2.8 $$K_{T}=\frac{\text{d}\sigma_{T}}{\text{d}\delta_{T}},$$

where *δ_N_* and *δ_T_* are the normal and tangential stresses, respectively. It is often interpreted that *δ_N_* is the approaching distance between the average height of the roughness asperities distributed on the two contacting surfaces, and *δ_T_* is the relative distance in the tangential direction between the asperities on the two contacting surfaces (figure 4) [32–36]. In the case of contact stiffness evaluated by ultrasound, both stress and displacement are interpreted as the values in the nominal (apparent) contact area of the imperfect contact surface where the ultrasound is incident. As shown in the equation below, the reflectivity is represented by the ratio between the intensities of the reflected wave when a load is applied and that when no load is applied:

2.9 $$R=\frac{H}{H_{0}},$$

where *H* and *H*_0_ are the reflected wave intensity with load applied and no contact, respectively. Here, ‘intensity’ is defined as a peak-to-peak magnitude of the reflected wave in the time domain, as described in §2d. The intensity of the wave reflected by the interface between steel and air when no load is applied is assumed to be equivalent to the intensity of the incident wave. It can be used as a simple and effective method for eliminating the influence of the inherent characteristics of the probe and attenuation/dispersion of the ultrasound.

In this study, the ‘imperfect’ interface is considered as a spring and the stiffness of the spring (contact stiffness) is evaluated, as shown in figure 4. The degree of imperfection of the interface is of course strongly influenced by the size and distribution of the asperities, i.e. roughness of the two contacting surfaces. As the roughness deforms micro- and macroscopically due to the stresses created at the interface, the contact stiffness will change as well. Many studies have already been carried out to study the influence of surface asperities on contact stiffness and their realistic behaviour including their deformation with normal load using ultrasound [37,38]. More recently, the contact stiffness of interfaces loaded with tangential forces has been measured to understand the dynamic frictional behaviour [28,39]. Therefore, in this study, dynamic measurements of contact stiffness were carried out in order to understand the changes in contact conditions due to differences in roughness under high-pressure friction conditions simulating a wheel–rail contact.

Some of the authors repeatedly measured the contact stiffness distribution in the normal direction in the contact area of the wheel–rail under rolling–sliding conditions using ultrasonic waves and reported that a linear relationship was observed between the contact stiffness in the normal direction and the friction coefficient [27]. It was assumed that the contact stiffnesses in the normal and tangential directions were similar, but it is not clear whether the contact stiffnesses are actually similar or not. For this reason, contact stiffness measurements in three different directions, normal and two tangential directions, slip direction and perpendicular to the slip direction, were conducted in this study.

### (c) Specimens

The top and the bottom specimens were made from ER8 (EN13262:2009) wheel material and R260 (EN13674-1:2011) rail material, respectively. The hardness values in HV(5) were 267 for the ER8 wheel (top) specimen and 285 for the R260 rail (bottom) specimen. The hardness values were the average for 5 measurements. The measurements were conducted using a Mitutoyo hardness testing machine HV-110 and test force was 5 kgf.

**Figure 5.**
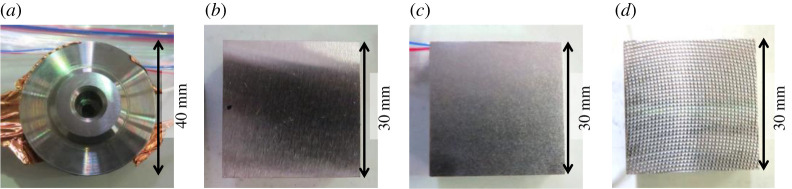
The appearance of specimens on the contact side. (*a*) Top specimen. (*b*) Bottom specimen: low roughness (ground). (*c*) Bottom specimen: medium roughness (sandblasted). (*d*) Bottom specimen: high roughness (milled). (Online version in colour.)

The surface type of the top specimens remained constant for all tests and was achieved by grinding to get a repeatable surface finish for all tests, and the surface types of bottom specimens were varied between tests. Three different roughnesses were achieved by grinding, sandblasting and machining (milling), which will be referred to as low roughness, medium roughness and high roughness, respectively. The initial roughness profiles can be seen in §3d. Figure 5 shows the appearance of the specimen contact surfaces.

**Figure 6.**
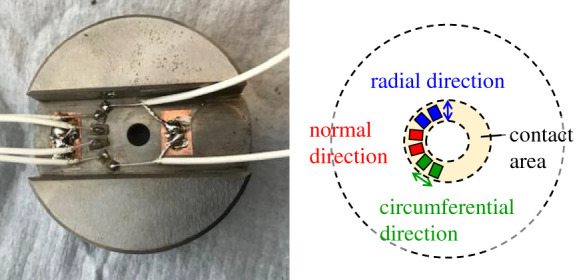
Piezoelectric elements attached on the back of the top specimen. (Online version in colour.)

For all the contact tests, specimens were soaked in 2-propanol and washed in an ultrasonic bath before the measurement. Miniature piezoelectric elements were bonded to the top specimen to generate ultrasonic waves that reflected off the contact interface and they also measure the reflected wave. Figure 6 shows the longitudinal and transverse piezoelectric elements which were attached on the back of the top specimen.

The longitudinal element oscillates a wave in which the displacement of the medium coincides with the direction of motion of the wave, while the transverse element oscillates a wave in which the displacement of the medium is perpendicular to the direction of motion of the wave. The longitudinal and transverse waves reflected on the interface contain information about the contact stiffness in the normal and tangential directions, respectively.

When the frictional force acts on the interface, the tangential contact stiffness may behave differently from normal contact stiffness. In particular, the contact stiffness in the tangential direction may also be different between the perpendicular (radial) direction and the slip (circumferential) direction. Therefore, it is important to evaluate the difference of contact stiffness in different directions so three directions of contact stiffness were measured in this study: normal, radial and circumferential.

Off course, it is possible that the friction conditions may be slightly different at each measurement position, but this should provide useful information on whether there are obvious differences between directions. Therefore, measurements were made in all three different directions. Taking into account the direction of polarization of the piezo element, the piezoelectric elements for measurement of the transverse waves were installed in directions parallel (circumferential direction) and perpendicular (radial direction) to the friction direction. The piezoelectric element was made of lead zirconate titanate (PZT) as a base material, with thicknesses of approximately 0.4 mm for longitudinal and approximately 0.2 mm for transverse. The PZT plate was cut into rectangles with a side length of about 1–3 mm. Piezoelectric elements with a central frequency of 5 MHz were used for the measurements of both longitudinal and transverse waves. In this test, a ‘Pitch-Catch’ method was employed in which different piezoelectric elements are used for activation and reception of the ultrasound.

Howard scanned the area of the ultrasonic wave emitted by the PZT which was directly attached to the test object as above, using a fine, ball-tipped probe fitted with a PZT for the receiver [40]. The results of mapping the signal amplitude showed that the effective ultrasound spread for the measurement did not deviate much from the size of the PZT attached to the test object, forming a concentrated area.

### (d) Test procedure

**Figure 7.**
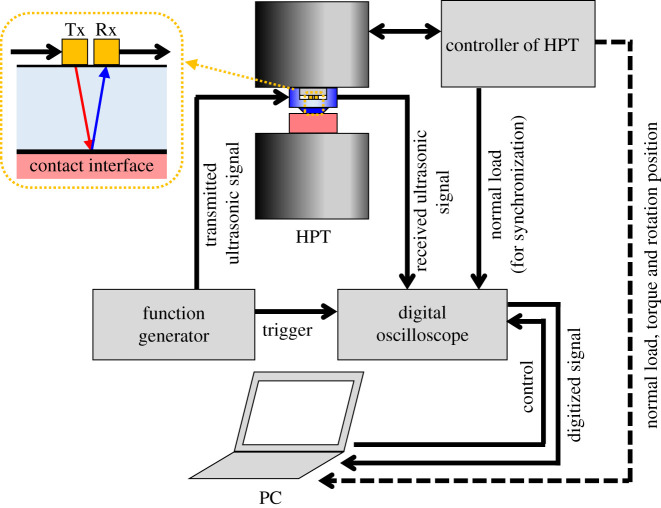
Schematic of whole experiment set-up. (Online version in colour.)

Figure 7 shows a schematic of the experimental set-up. A 20 V peak-to-peak, 5 MHz, 3-cycle, sine wave was generated by a function generator (TG5011A, AIM-TTI Instruments) and used to excite the piezoelectric elements on the actuator side and generate an ultrasonic wave. The ultrasonic waves then propagated to the contact interface, and the wave reflected from the interface was received by the piezoelectric elements on the sensor side. The received waveforms were digitized without any amplification using a digital oscilloscope (Picoscope 5000 series, PicoTech) and stored on a PC. The information from the HPT controller (normal load, torque and rotation position) was obtained off-line, but a part of the information (normal load) was shared by the oscilloscope and used to synchronize the ultrasonic and HPT data in post-processing.

**Figure 8.**
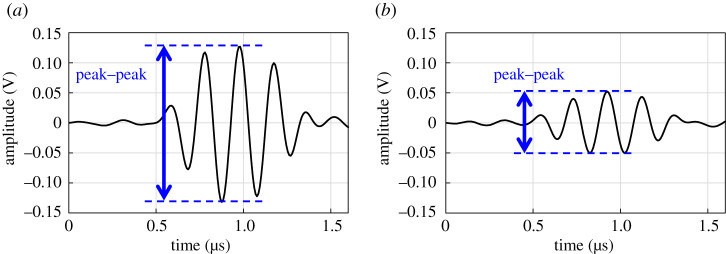
Examples of reflected waveforms of longitudinal wave after bandpass filter. (*a*) Without contact. (*b*) With contact. (Online version in colour.)

The measured reflection waveforms were bandpass filtered from 3 MHz to 7 MHz. Figure 8 shows examples of reflected waveforms of a longitudinal wave after bandpass filtering. Two cases are shown here, with and without contact. The values between the maximum and minimum peaks are used as the evaluation values in time domain.

**Figure 9.**
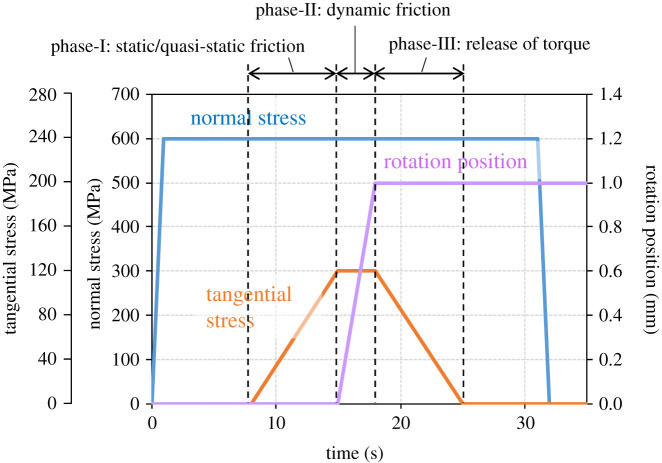
Schematic example of a loading cycle. (Online version in colour.)

Figure 9 shows a schematic example of a loading cycle. The test was conducted using the following procedure:

1. A normal stress is applied after inserting a pressure-sensitive paper between the specimens to check that the load is uniformly distributed on the contact area.
2. After making the specimens directly contact with each other, the normal stress is increased gradually to approximately 600 MPa.
3. While keeping the top specimen in the specified position, the tangential stress is applied. (Phase-I)
4. The bottom specimen is rotated. (Phase-II)
5. The tangential stress is gradually released. (Phase-III)
6. After releasing the torque, the specimens are separated.

During steps 2 to 6, the contact stiffnesses are measured by using ultrasound.

### (e) Numerical simulation

In order to verify whether changes in contact stiffness can occur in the test conditions, numerical contact simulations based on a boundary element method with the half-space approximation were conducted, and the results were compared with the experimental results. There are several methods to perform contact calculations using rough surfaces, such as assuming the asperity distribution to be an ideal distribution (e.g. Gaussian) [41,42] or transforming the distribution into a shape based on the measured roughness profile [43]. However, in this study, we adopted the method of bringing together the measured three-dimensional geometries. This method can be one of the most direct calculation methods because it applies real geometry.

The numerical calculations were conducted using three-dimensional surface topography data measured by a non-contact surface profiler (InfiniteFocus, Alicona) before test cycles. As a consequence of the complexity of the top specimen geometry, the result from the bottom specimen low roughness test was used instead. Both were ground finishes and were judged to have similar roughness profile, as shown in §3d.

The boundary element formulation for a normal contact converts the problem of finding displacement at the surface points given a set of pressures on each of the surface points, to a convolution between the surface pressures and a ‘short-form’ influence matrix which depends on the material properties of the surface and the discretization grid. Alternatively, this process can be thought of as a matrix multiplication between a vector of pressures and a `long-form’ influence matrix. In practice, the convolution method is almost always used as this allows the process to be accelerated by completing the convolution in Fourier space (if x(*t*) ⊛ h(*t*) = y(*t*), then X(*f*)H(*f*) = Y(*f*)) [44].

For contact between two surfaces, these influence matrices can be summed to give the total deformation of the pair of surfaces due to a set of mutual loads. In general, for a rough surface contact the inverse problem is solved, finding the loads required to produce deformation in the surfaces so that the surfaces do not penetrate each other. The domain of this solution is confined to areas of the surface where this load is positive. This can be solved by a suitable conjugate gradient method.

In this work, the domain of the solution is further confined to areas where the normal surface pressure is less than a limiting pressure, based on the material hardness as given by [45]. Areas which contact, but would require a load larger than this critical load to not penetrate, are allowed to penetrate and the critical load is applied. This constraint has been added to the BCCG method given in [46]. After each time step of the simulation this penetration is removed as plastic wear.

When the normal contact problem has been solved, the contact stiffness must be found. At any point, the response of the system to small perturbations of load can be thought of as linear providing the perturbation is not large enough to change the domain of the solution (the points of the discrete surface profile which are in contact). In practice, we can set the domain of the solution, remove the minimum and maximum load constraints and find the pressures on the currently contacting nodes required to produce a unit deformation of those nodes. In the equation below these pressures are *L*^1^, the displacement vector caused by these pressures is *D*^1^ and the influence matrix is *M*. The sum of these pressures is the contact stiffness as given by [47]. However, this total stiffness includes all of the deformation from the infinite half-space as well as the contact region:

2.11 $$\begin{array}{rl} M⊛L^{1} & =D^{1}, \end{array}$$

2.12 $$\begin{array}{rl} D_{i}^{1} & =1:i\in \text{Conact Area, } \end{array}$$

2.13 $$\begin{array}{rl} L_{i}^{1} & =0:i\notin \text{Conact Area} \end{array}$$

2.14 $$\begin{array}{rl} \text{and}\;\;k_{\text{all}} & =\frac{\Delta l}{\Delta d}=\frac{\sum_{i=0}L_{i}^{1}a}{1}=a\underset{i=0}{\sum }L_{i}^{1}. \end{array}$$

In the ultrasound community, it is common to define the contact stiffness as the load per unit gap closure. This is a different but related quantity. Previous studies have found this quantity by perturbing the system with a small load and directly measuring the change in gap height from the simulation result [48]; however, this method is error prone. Below it is shown that the gap definition of contact stiffness can be linked to the total stiffness:

2.15 $$\begin{array}{rl} \underset{\Delta l\to 0}{\lim}\frac{\Delta l}{\Delta \bar{G}} & =k_{\text{gap}}, \end{array}$$

2.16 $$\begin{array}{rl} \Delta G_{i} & =G_{i}^{0}-G_{i}^{\Delta l}=G_{i}^{0}-(G_{i}^{0}+D_{i}-\Delta d) \end{array}$$

2.17 $$\begin{array}{rl} \text{and}\;\;\Delta G_{i} & =D_{i}-\Delta d, \end{array}$$

where *G_i_* is the gap vector, with superscripts 0 and Δ*l* meaning the original gap and the gap after the application of a small load Δ*l*, *D_i_* is the displacement vector caused by this small load and Δ*d* is the approach of distant points in the solids caused by this small load. The overbar signifies the mean value of a vector. As the system is linear, the following can be written:

2.18 $$\begin{array}{rl} \Delta d & =\frac{\Delta l}{k_{\text{all}}}, \end{array}$$

2.19 $$\begin{array}{rl} D_{i} & =\frac{D_{i}^{1}\Delta l}{a\sum_{i=0}L_{i}^{1}}, \end{array}$$

2.20 $$\begin{array}{rl} \Delta G_{i} & =\left(\frac{D_{i}^{1}-1}{k_{\text{all}}}\right)\Delta l \end{array}$$

2.21 $$\begin{array}{rl} \text{and}\;\;k_{\text{gap}} & =\frac{\Delta l}{\Delta \bar{G}}=\left(\frac{k_{\text{all}}}{\bar{{\bar{D}}^{1}-1}}\right). \end{array}$$

Clearly, when the entire surface is in contact *D_i_*^1^ = 1: ∀*i* and this gap stiffness tends to infinity as expected. This method can also be used to calculate the contact stiffness in either the loading or unloading directions; by excluding nodes which are at the maximum pressure the loading stiffness will be found.

The normal contact equations were solved for a 1024 by 1024 grid, using the experimentally measured surface profiles shown above. The code used to solve the models has been added to ‘Slippy’ (v. 0.1.4), an open-source contact modelling package. The code used to generate these models is provided in the additional material.

## 3. Results

### (a) Change in the contact stiffness during normal force loading

**Figure 10.**
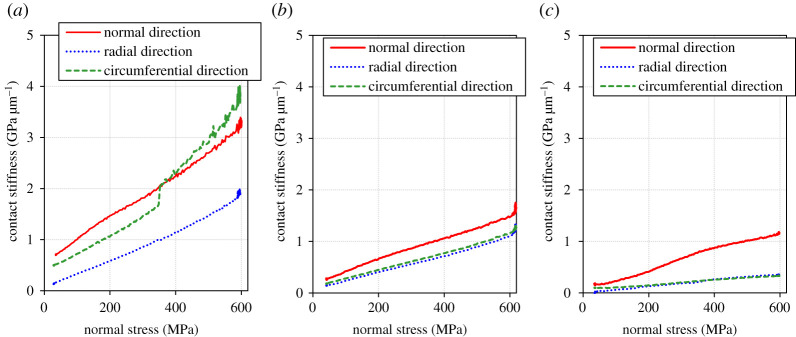
Relationship between the normal stress and the contact stiffness which was measured during loading (step 2 in test procedure). (*a*) Low roughness, (*b*) medium roughness and (*c*) high roughness. (Online version in colour.)

Figure 10 shows the relationship between the normal stress and the contact stiffness which was measured during normal loading (step 2 in test procedure). It indicates that the contact stiffness has a positive correlation with the normal stress on the contact area for all the directions and all the different roughness cases. This result coincides with many previous reports [15,33,37]. Figure 10*a* shows a sharp increase in contact stiffness of the circumferential direction between 300 and 400 MPa in the low roughness condition. Although physical verification is not possible at this moment, a local variation in contact pressure (e.g. sudden increases in contact pressure where it was not previously high) may improve the conformity between the two bodies rapidly. When considering a Hertzian contact, it is known that a smooth surface has a higher maximum pressure and a smaller contact area than a rough surface [49,50]. Considering the above, if the elastic deformation of the specimen causes a non-uniformity in the pressure distribution in the contact surface, the degree of non-uniformity may be more pronounced for the lower roughness. This possibility is also supported by the fact that local contact is more pronounced in lower roughness conditions, as shown in §3d (figure 15).

### (b) Change in the contact stiffness under tangential load

**Figure 11.**
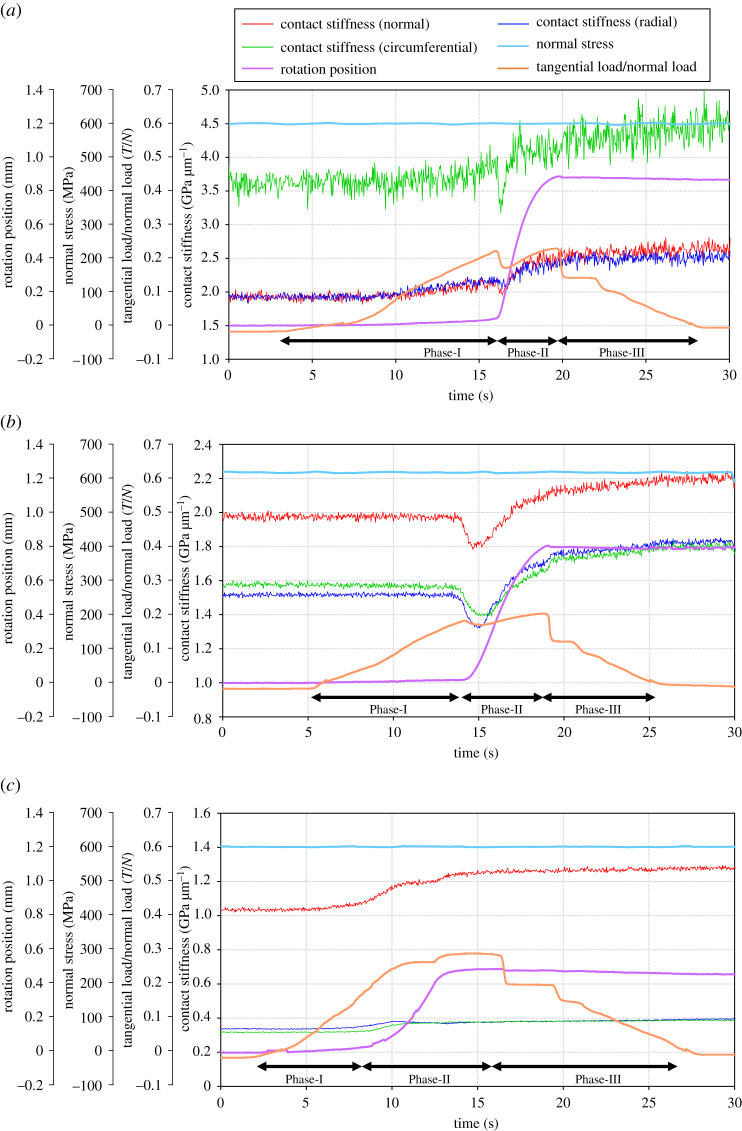
Changes in the parameters with testing time. Parameters: contact stiffness, *T*/*N*, normal stress, rotation position (steps 3 to 5 in test procedure). (*a*) Low roughness, (*b*) medium roughness and (*c*) high roughness. (Online version in colour.)

Figure 11 shows the changes in the contact stiffness, tangential load/normal load (hereafter referred to as *T/N*), normal stress and rotation position with the time, obtained from steps 3 and 4 of the test procedure. Here, the positive pressure value is defined as the pressure in the compression direction for normal stress. In Phase-I, there was little or no sliding while *T/N* increased. For this period, the specimens were assumed to be in a static or a quasi-static contact condition except for the possibility of the rig being slightly twisted, and the contact stiffnesses remained at almost constant level or slightly increased. In Phase-II, the rotation position rapidly increased. At the same time, the contact stiffness rapidly decreased at the onset of slip, then slightly increased after that in the case of low and medium roughness. On the other hand, in the case of the highest roughness, the contact stiffness no longer showed the dip and the rotation curve rises gently with *T*/*N*. In Phase-III, though the rotation stopped and *T*/*N* decreased as the tangential stress was released, the contact stiffness kept a constant level or slightly increased. At the same time, a slight restoration of the rotational position was found. It is considered that a spring-back (relaxation) at the contact interface occurred.

In all cases of roughness, though the *T*/*N* decreased to zero when the tangential stress was released after rotation, *T*/*N* showed a negative value before Phase-I. It is considered that a slight misalignment in the horizontal direction between the specimen and the testing equipment generated tiny slip even when only normal stress was applied.

**Figure 12.**
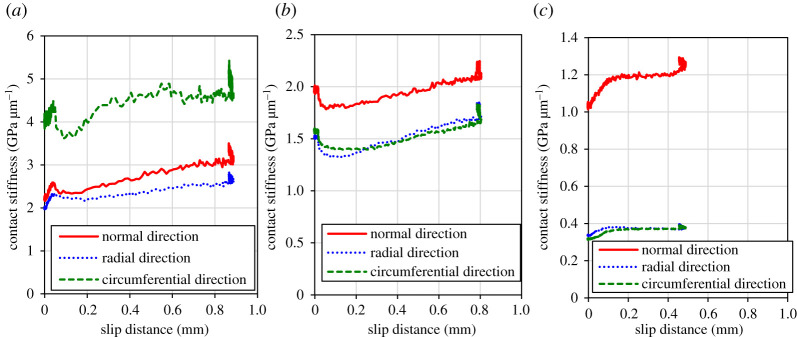
Relationships between the slip distance and the contact stiffness. (*a*) Low roughness, (*b*) medium roughness and (*c*) high roughness. (Online version in colour.)

Figure 12 shows the relationship between the slip distance and the contact stiffness while the tangential stress was being applied. This figure shows that for low and medium roughness, there is a positive correlation between the slip distance and the contact stiffness. This may be due to wear and deformation of the initial asperities, as discussed in §4c, which has increased the contact area and therefore the contact stiffness. At high roughness, the contact stiffness increased in the early stages of slip, but then the stiffness curve became flat with slip. It is thought that the macroscopic asperities were embedded in a smooth surface, making the contact more conformable and stiffer.

### (c) Change in the contact stiffness during normal force unloading

**Figure 13.**
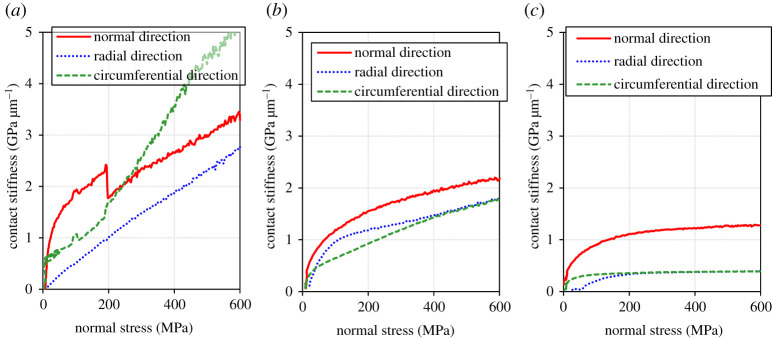
Relationship between the normal stress and the contact stiffness which was measured during unloading (step 6 in test procedure). (*a*) Low roughness, (*b*) medium roughness and (*c*) high roughness. (Online version in colour.)

Figure 13 shows the relationship between the normal stress and the contact stiffness which was measured during the normal stress unloading (step 6 in test procedure). The overall contact stiffness was higher than that of loading, but this would be due to the increase in contact stiffness with slip, as shown in figure 12. It was found that the change of contact stiffness follows a different path in the procedure of loading and unloading. Although the contact stiffness during loading showed a more linear-like increase, it showed a square root-like function or an asymptote with the decrease of normal stress in the procedure of unloading. Drinkwater *et al.* and Dwyer-Joyce *et al.* conducted loading–unloading cycles measuring contact stiffness and reported hysteresis trends [37,38], and they indicated that a possible influence of the plasticity of asperities occurs in the loading cycle. Although figure 13*a* shows a sharp decrease in contact stiffness of normal direction around 300 MPa in the low roughness condition, this may have been also due to a local variation in contact pressure as in figure 10*a*.

### (d) Change in the roughness profile after the cycle

**Figure 14.**
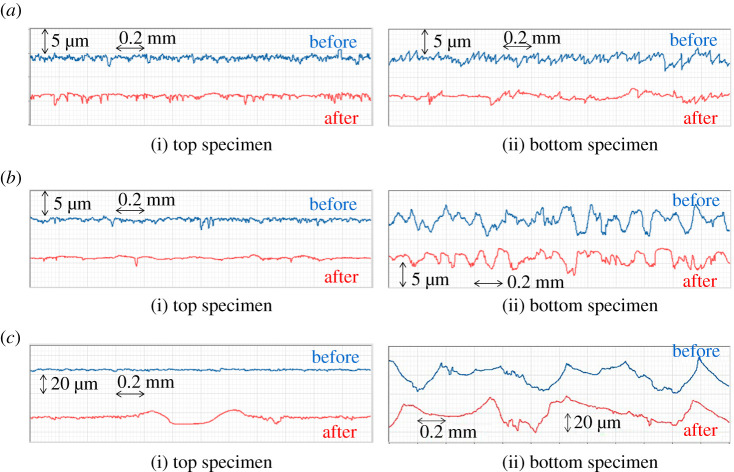
Roughness profiles of the specimens in circumferential direction before and after the tests. (*a*) Low roughness, (*b*) medium roughness and (*c*) high roughness. (Online version in colour.)

Figure 14 shows the roughness profiles of the specimens measured before and after the tests in circumferential direction which were measured using a contact roughness meter (Mitutoyo Surftest SJ-210) with a cut-off of 0.8 mm and a length of 2.4 mm. The measurement positions of the roughness profiles were not exactly the same before and after the tests. However, as the pre-test specimens were prepared in the workshop, the roughness profile was consistent. It is also clear that the geometry characteristics have not changed significantly over the 2.4 mm measurement length, both before and after the test. It is therefore considered that these roughness profiles provide a sufficiently representative trend of the profile change.

Comparing the roughness profiles, it was found that the shape of the summits of the asperities has been rounded and flattened after the test in the cases of low and medium roughness. The roughness profiles of the top specimen in the case of high roughness show large deformations after the test. It is considered that the asperities of the bottom specimen were stuck and transcribed to the top specimen.

**Figure 15.**
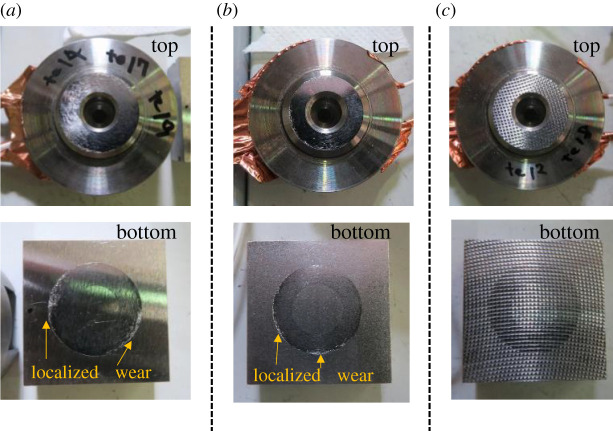
Photographs of the contact surfaces of the specimens after the tests. (*a*) Low roughness, (*b*) medium roughness and (*c*) high roughness. (Online version in colour.)

Figure 15 shows photographs of the contact surfaces of the specimens after the test. For all roughness conditions, a circular contact area is clearly shown on the surface of the bottom specimen after the test. At the high roughness condition, the surface of the top specimen after the test shows a dotted pattern, which is thought to be the imprint of the asperities of the bottom specimen. At lower roughness levels, localized wear marks were observed at the outer circular edge of the contact area, but not at higher roughness levels.

**Figure 16.**
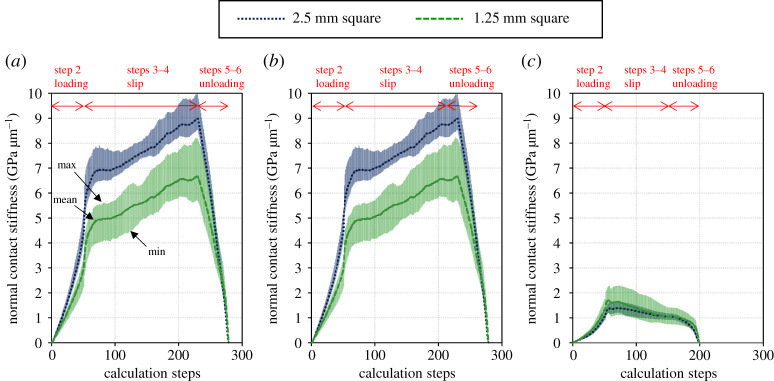
Changes in normal contact stiffness simulated by the same test cycle as in the experiment. (*a*) Low roughness, (*b*) medium roughness and (*c*) high roughness. (Online version in colour.)

### (e) Comparison of experimental and numerical simulation results

Figure 16 shows the changes in normal contact stiffness simulated by the same test cycle as in the experiment. The real contact area is not evenly distributed across the apparent contact area. Close to the edges, the local pressure is higher, leading to a higher real contact area. This can be seen from the surface condition of the specimen after the test, shown in figure 15. As such, in order to be compared to the measured results, the domain of the contact stiffness calculation is reduced to a patch in the centre of the apparent contact area.

The size of this patch greatly influences the result of the calculation; larger patches include more of the edge and give a higher contact stiffness. This is most pronounced for smoother surfaces. Because of this, results from two different sized areas are shown: 2.5 mm square and 1.25 mm square. These sizes represent the projected size of the sensor if the emitter is considered as an area, respectively. Results shown are averaged from 32 measurement regions each offset in the periodic grid direction by 32 grid points (0.16 mm) to account for the random variation in the surfaces. The figure also shows the range of maximum and minimum values of the 32 numerical values as the degree of fluctuation.

The contact stiffness tends to be higher for a 2.5 mm square than for a 1.25 mm square. As mentioned above, this is because the greater area is more affected by the locally higher contact stiffness at the edge. In the following sections, the experimental and numerical results of the individual steps will be compared.

**Figure 17.**
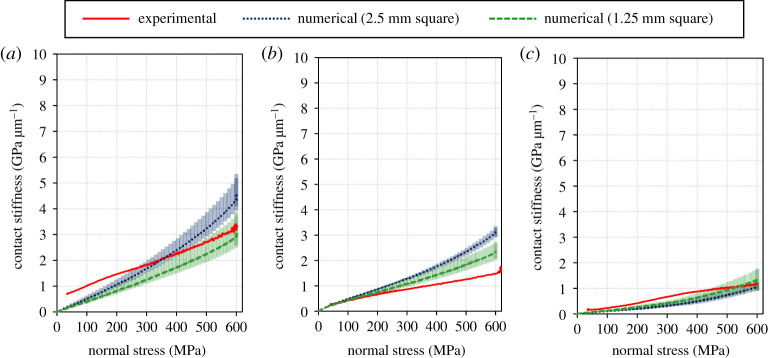
Contact stiffnesses in normal direction during loading estimated by the numerical calculation compared with those measured during the experiment. (*a*) Low roughness, (*b*) medium roughness and (*c*) high roughness. (Online version in colour.)

Figure 17 compares the contact stiffnesses in the normal direction during loading (step 2) estimated by the numerical calculation with those measured by experiment. For all roughness conditions, the numerical simulations reproduced the increasing trend of normal contact stiffness with increasing stress.

**Figure 18.**
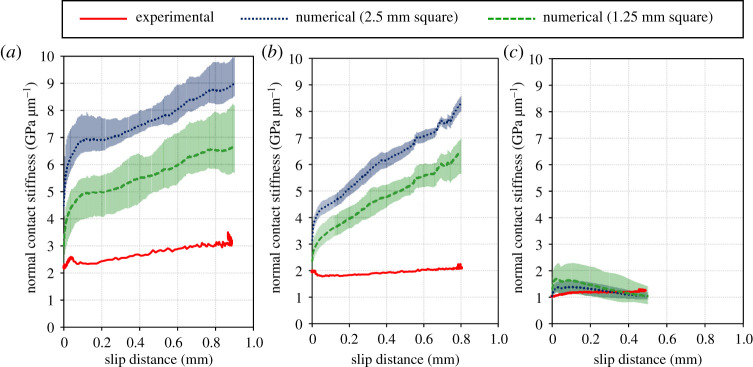
Contact stiffnesses in normal direction during slip estimated by the numerical calculation compared with those measured during the experiment. (*a*) Low roughness, (*b*) medium roughness and (*c*) high roughness. (Online version in colour.)

Figure 18 compares the contact stiffness in the normal direction estimated by the numerical calculation during slip (steps 3–4) with the experimental one. For the two smoother surfaces, the numerical prediction rapidly increases during slip as a material is removed to account for plastic deformation. As shown, this leads to a large over prediction by the end of the slip. For the higher roughness, this effect is counteracted by the surfaces becoming less conformal.

**Figure 19.**
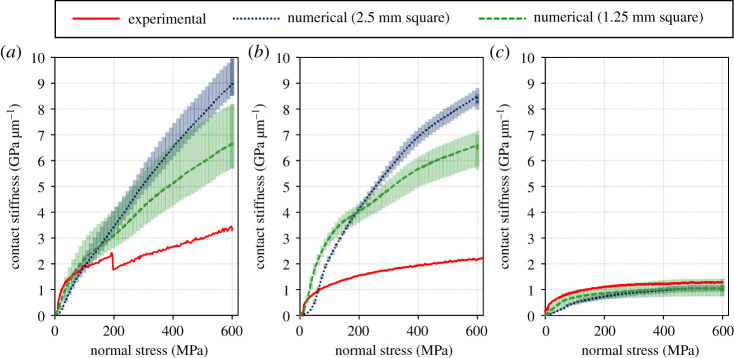
Contact stiffnesses in normal direction during unloading estimated by the numerical calculation compared with those measured during the experiment. (*a*) Low roughness, (*b*) medium roughness and (*c*) high roughness. (Online version in colour.)

Figure 19 compares the contact stiffnesses in the normal direction during unloading (steps 5–6) estimated by the numerical calculation with those measured during the experiment. As in the slip, and for the same reasons, the magnitude of the stiffness is higher for the model in all but the roughest case. However, the form of the result is generally well captured, and this result is not simply the opposite of the loading curve as the surface becomes more conformal due to plastic deformation.

## 4. Discussion

### (a) Relationships between the work energy and contact stiffness

Figure 12 shows the increase of contact stiffness around the end of the rotation, even though little slip has occurred. To investigate this phenomenon from another aspect, the work by the friction was evaluated. The work, *W*, was calculated by the following equation:

4.1 $$W=\int_{L_{0}}^{L_{1}}|S|\;\text{d}L,$$

where *L* is the accumulation distance and calculated by the following equation:

4.2 $$L=\int_{t_{0}}^{t_{1}}|l|\;\text{d}t,$$

where *l* is the rotation distance, *t*_0_ is the time when the tangential stress was applied and *t*_1_ is the time when the tangential stress was released.

**Figure 20.**
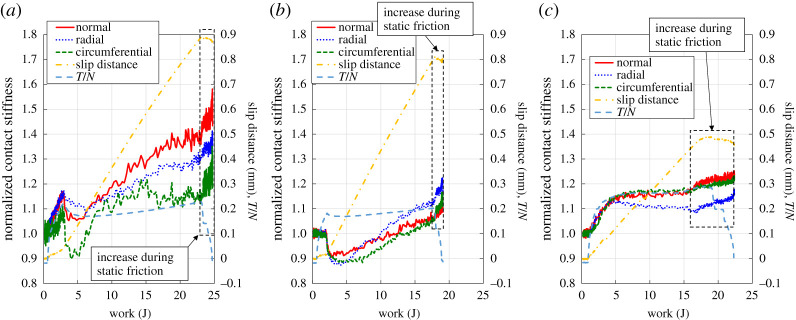
Relationships between the work and the normalized contact stiffness with the slip distance and *T/N*. (*a*) Low roughness, (*b*) medium roughness and (*c*) high roughness. (Online version in colour.)

Figure 20 shows the relationship between the work and the normalized contact stiffness with the slip distance and *T*/*N*. It should be noted that the contact stiffness was normalized as the initial value by the contact stiffness before the application of tangential stress. It is found that the normalized contact stiffness basically increased with the work for all roughnesses. However, it dropped around 2–5 J in the cases of low and medium roughness before rising again. This period is relevant to the beginning of Phase-II, the dynamic friction. On the other hand, there was no drop in the case of high roughness. Pesaresi *et al.* [28] also reported a decrease of the contact stiffness after a macro-slip. It is thought that the accumulation of work energy caused micro destruction of the adhesive interface and it led friction to drop, before rising again as new asperity junctions began to form in the cases of low and medium roughness. On the other hand, in the case of high roughness, it is thought that the plastic deformation of the asperities preceded the adhesion, and it suppressed the destruction of the interface.

It should be noted that for all roughness conditions the contact stiffness continues to increase while the apparent increase in slip distance ends and the *T*/*N* decreases. This may be due to the accumulation of work energy caused by the slight spring-back and residual tangential stress even in the static friction condition. Fantetti *et al.* [39] also reported an increase in ultrasound transmission (contact stiffness) under static friction (stick) conditions. They indicated that this is a consequence of junction growth as proposed by Tabor [51] and ageing, so-called time-dependent material creep [52], which could be adopted here.

### (b) Fluctuation of numerical results and differences with experimental results

The comparison between the experimental and modelled results shows relatively good agreement during the loading step of the experiment. During the sliding step, the deformation and wear of the surface are not captured well by the simple wear model applied, and consequently the modelled results from this and the subsequent unloading steps are generally poor. The range of values which can be produced by the model is relatively large, in the worst case approximately 50% of the maximum value. This high variation is a result of the application of different assumptions of how the ultrasound transducer should be treated and natural variation associated with the random nature of surface geometry. Agreement between the model and the experiment was best for the highest roughness case. In the model, this case also showed the lowest random variation between sampled areas and the least dependence on the size of the area used for the contact stiffness measurement. Further inspection of the results from the highest roughness model showed less pronounced edge effects than seen with either of the lower roughnesses. This is in line with the prediction made in §3a that the larger the roughness, the more gradual the local pressure distribution, as shown in previous literature [49,50].

The variation between different sampled areas of the same size is a natural result of the semi-random geometry of surfaces, while the variation between different size domains is a result of edge effects which are present both in the model and the real world. The difference between different size areas is large, and unfortunately, there is currently no consensus on what the correct area is. As the ultrasound wave is not uniform on incursion with the contact surface, it is additionally possible that there is no simple size of area which can be used.

### (c) Schematic representation of interfacial phenomena

**Figure 21.**
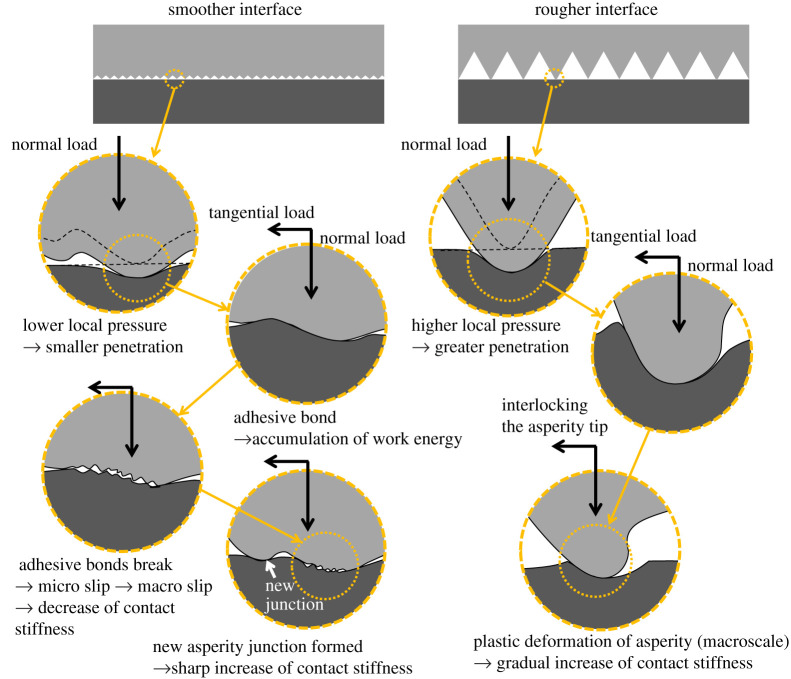
Schematic representation of the proposed mechanism of the change at the interface when the dynamic friction occurs. (Online version in colour.)

Figure 21 shows a schematic representation of the proposed mechanism for the change at the interface when the dynamic friction occurs. In the case of low/medium roughness, the summits of asperities are plastically deformed and make adhesive contacts at individual bonds due to the material similarity of the two bodies in contact. As the tangential load is applied and the Coulomb limit is approached, interfacial fracture begins to occur at each bond [52], and friction and stiffness simultaneously drop. This fracture is thought to occur microscopically at first, but quickly propagates to the entire interface as a macro-slip [53]. With macro-slip, new asperity junctions form (un-deformed asperities, previously not in contact), and stiffness begins to rise. Fantetti *et al.* [39] have conducted the test of high-frequency shear vibration and investigated the interface condition using ultrasound and reported the reduction of ultrasound transmission (reduction of contact stiffness). They attributed this to the occurrence of interfacial separation due to slippage, which will also be applicable to the phenomenon in this study.

On the other hand, in the case of high roughness, macroscale effects (i.e. each large machined peak) dominate as opposed to individual asperities (microscale). The summits of asperities also conformed, but the penetration of asperities is greater than the smoother interface because of the higher local pressure [54]. With the application of tangential load, the asperities plastically deform rather than break bonds owing to the interlocking of the summits due to penetration and the greater heights of asperities. In the field of metal forming, it is widely known that bulk plastic deformation occurs during shearing as the contact pressure increases [55,56]. Considering the greater asperity as a separated bulk body, it is possible that the plastic deformation of the asperity took precedence over the micro-slip. Hence, the contact stiffness gradually increases as a consequence from static/quasi-static friction to the dynamic friction. The bottom specimen in the high roughness condition was machined (milled) at a pitch of 0.8 mm, resulting in the formation of peaks at this pitch. This can be seen in figure 14*c*. The slip distance for the high roughness condition was about 0.5 mm (figure 11*c*), which is smaller than the spacing of the asperities. As the asperity pitch is sufficiently long in relation to the slip distance, plastic deformation may continue throughout the entire slip process. Therefore, asperity breakage (wear) and the formation of new contacts, as in the low/medium roughness condition, would be less likely to occur and the increase in contact stiffness could be reduced.

### (d) Relationships between the tangential stress and contact stiffness

**Figure 22.**
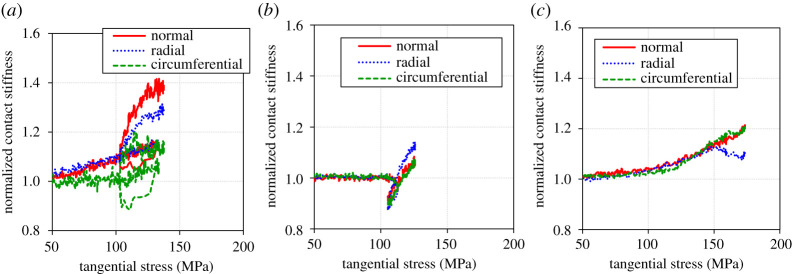
Relationships between the tangential stress and normalized contact stiffness from quasi-static to dynamic friction. (*a*) Low roughness, (*b*) medium roughness and (*c*) high roughness. (Online version in colour.)

Figure 22 shows the relationship between the tangential stress and the normalized contact stiffness from quasi-static to dynamic friction. The contact stiffness was normalized as the initial value by the contact stiffness before the application of tangential stress. In the low and the medium roughness condition, the contact stiffness was partly below one because there was a large drop in contact stiffness at the beginning of slip, as shown in figure 12. It was found that most of those increased with the tangential stress as well as the normal stress dependency in figures 10 and 13. This phenomenon is interesting because it shows a cycle between the application of tangential stress and the change in contact condition:

— Tangential stress is applied.
— The asperity deforms until it can withstand the tangential stress and the contact stiffness increases (interface becomes stiffer).
— The tangential stress is increased and the contact stiffness is increased again.

In other words, this is the initial stage of the running-in process, where the interface becomes harder and more resistant to tangential displacement as the conformity of the interface increases [26,27]. The dependency on tangential stress showed more scattering compared with the normal stress. It is thought that the change of surface asperities at slip interface is more complicated than the purely compressed interface which was constrained in the loading direction, because the friction joints could separate with relative motion.

### (e) Directional dependence of contact stiffness at the friction interface

Throughout all the results, there was little difference in the contact stiffness between the directions, normal, radial and circumferential. This can be seen from the fact that there is no difference between the radial and circumferential directions in the roughness profile after the test in the additional data (Dryad). In particular, figures 11 and 12 compare the change in contact stiffness in each direction under tangential load, which was almost uniform for all roughness conditions. Based on Nagy's assumption that the ratio of tangential to normal stiffness is affected by the aspect ratio of the cavities [57], it could be said that the shape of the cavities did not change significantly under the friction conditions of the present study, which simulated wheel–rail contact conditions. In other words, these results showed that the behaviour of the tangential contact stiffness can be roughly predicted by measuring the normal contact stiffness. However, such behaviour can be dependent on the material, roughness and friction conditions. In particular, the short slip distance of less than 1 mm in this case may have prevented the anisotropy from becoming apparent, which needs to be investigated further.

## 5. Conclusion

Aiming to understand the influence of the roughness on the dynamic friction behaviour between the wheel and rail, ultrasonic reflectometry was applied to the HPT test approach, and *in situ* evaluation of the contact condition was carried out. From the results of evaluation, the following conclusions can be drawn:

(1) The application of the ultrasound reflectometry to the HPT test approach enabled *in situ* evaluation of the friction interface under extremely high contact pressure and gave the information about continuous change of interfacial topographies and contact stiffnesses with friction. It is thought that this technique can be applied not only to the wheel–rail interface, but also to general high contact pressure interfaces.
(2) The effect of roughness on the change in contact stiffness during the transition from static and quasi-static friction modes to dynamic friction mode could be evaluated. In the low roughness condition, a temporary decrease and subsequent increase in contact stiffness was observed when macroscopic slips occurred, suggesting a breakdown of the interface and the formation of new asperity junctions. In the high roughness conditions, the effect of plastic deformation of the macroscopic roughness asperities was pronounced. The contact stiffness gradually increased with tangential stress loading.
(3) Numerical calculations using real surface topography could reproduce the change in contact stiffness under a series of test conditions: loading, slip and unloading. Particularly for the high roughness condition, the experimental and numerical results were in good agreement and the fluctuation due to the different calculation domains was small.
(4) There was a positive relationship between the slip distance and the contact stiffnesses. It was considered to indicate that the wear and plastic deformation of the asperities progressed and conformed along with the increase of the slip distance. The dependency of the direction on the contact stiffness was not significant and they were considered to change almost uniformly.

## Data accessibility

See https://doi.org/10.5061/dryad.12jm63z01.
